# Adult-Like Anti-Mycobacterial T Cell and *In Vivo* Dendritic Cell Responses Following Neonatal Immunization with Ag85B-ESAT-6 in the IC31® Adjuvant

**DOI:** 10.1371/journal.pone.0003683

**Published:** 2008-11-10

**Authors:** Arun T. Kamath, Anne-Françoise Rochat, Mario P. Valenti, Else Marie Agger, Karen Lingnau, Peter Andersen, Paul-Henri Lambert, Claire-Anne Siegrist

**Affiliations:** 1 World Health Organization Collaborating Center for Vaccinology and Neonatal Immunology, Departments of Pathology-Immunology and Pediatrics, University of Geneva, Geneva, Switzerland; 2 Adjuvant Research, Department of Infectious Disease Immunology, Statens Serum Institut, Copenhagen, Denmark; 3 Intercell AG, Vienna, Austria; Karolinska Institutet, Sweden

## Abstract

**Background:**

With the exception of some live vaccines, e.g. BCG, subunit vaccines formulated with “classical” adjuvants do not induce similar responses in neonates as in adults. The usual neonatal profile is characterized by lower levels of TH1-associated biomarkers. This has hampered the development of new neonatal vaccines for diseases that require early protection. Tuberculosis is one of the major targets for neonatal immunization. In this study, we assessed the immunogenicity of a novel candidate vaccine comprising a mycobacterial fusion protein, Ag85B-ESAT-6, in a neonatal murine immunization model.

**Methods/Findings:**

The Ag85B-ESAT-6 fusion protein was formulated either with a classical alum based adjuvant or with the novel IC31® adjuvant. Following neonatal or adult immunization, 3 parameters were studied *in vivo*: (1) CD4^+^ T cell responses, (2) vaccine targeting/activation of dendritic cells (DC) and (3) protection in a surrogate mycobacterial challenge model. Conversely to Alum, IC31® induced in both age groups strong Th1 and Th17 responses, characterized by multifunctional T cells expressing IL-2 and TNF-α with or without IFN-γ. In the draining lymph nodes, a similarly small number of DC contained the adjuvant and/or the antigen following neonatal or adult immunization. Expression of CD40, CD80, CD86 and IL-12p40 production was focused on the minute adjuvant-bearing DC population. Again, DC targeting/activation was similar in adults and neonates. These DC/T cell responses resulted in an equivalent reduction of bacterial growth following infection with *M. bovis* BCG, whereas no protection was observed when Alum was used as adjuvant.

**Conclusion:**

Neonatal immunization with the IC31®- adjuvanted Ag85B-ESAT-6 subunit vaccine elicited adult-like multifunctional protective anti-mycobacterial T cell responses through the induction of an adult pattern of *in vivo* DC activation.

## Introduction

Tuberculosis (TB) continues to be a global disease burden despite the widespread use of Bacillus Calmette Guerin (BCG) immunization (www.who.int/mediacentre/factsheets/fs104/en). Considerable efforts are aiming at the development of novel, safer and more efficacious vaccines against tuberculosis [1], [2]. A major limitation towards the achievement of this goal is a lack of reliable biomarkers of protective immunity against *M. tuberculosis* (Mtb). Both CD4^+^ and CD8^+^ T cells may contribute to protection, a central role for CD4^+^ T cells being suggested by the disease patterns in HIV-1 infected patients [3]. A critical function for anti-mycobacterial effectors is to produce type 1 cytokines, as highlighted by the severity of mycobacterial infections in children with genetic mutations in the IL-12/IFN-γ axis [4], [5] or by the high rate of Mtb reactivation and disease progression in patients treated with TNF-α inhibitors [6]. Multifunctional T cells concomitantly expressing several cytokines are thought to play a crucial role in protection against various infections [7], [8], and this may also apply to tuberculosis.

Exposure to Mtb may occur very early in life and infections with Mtb are frequently severe in infants and young children whose immature immune system fails to limit bacterial spread [9]. Therefore immunization strategies against TB should include the neonatal induction of potent anti-mycobacterial responses and to prove safety of such neonatal strategies. Currently, BCG vaccination is quite effective (approximately 80%) in human infants [10]. It may induce adult-like IFN-γ responses [11], [12], probably as a result of potent DC activation as BCG enhanced responses to a simultaneously administered hepatitis B vaccine [13]. However, although it was recently demonstrated that BCG elicits infant T cells with complex cytokine and phenotypic profiles [14], [15], no specific pattern distinguishing BCG-protected from non-protected children has yet emerged. Major BCG-related issues are (1) a limited persistence of the protective efficacy and failure to protect against pulmonary disease, (2) negative interference by previous exposure to environmental mycobacteria, (3) safety concerns in HIV-1 infected children. This emphasizes the need for safer and more efficacious infant TB vaccines.

Novel TB vaccines have been recently developed and a few have already entered into clinical trials which will define their safety and immunogenicity in naïve or previously exposed adults [1]. At this stage, predicting which of the novel candidates might also prove immunogenic in human infants will be largely empirical and lead to difficult “go-no go” decisions. The general objective of our studies is to generate preclinical evidence supporting the decision process for further vaccine development in children and infants. There is ample evidence that mice may not be reliably used to predict human vaccine efficacy. However, the main stages of immune maturation are sufficiently well conserved between humans and mice for specific neonatal animal models to accurately predict whether infant B and T cell response patterns will compare to those elicited in immunologically mature hosts [16]–[19]. It is of interest that both human and murine neonates exhibit limited IFN-γ expression capacity and limited Th1 responses that likely reflect differences in neonatal and adult DC activation profiles [20]. Aluminium salts, the only adjuvants currently licensed for use in infants, exacerbate the Th2-like profile of responses [21]–[23]. Remarkably, these neonatal limitations can be overcome by some specific vaccines and/or through appropriate DC activation signals [11], [24], [25]. It appears of importance *a priori* that new TB vaccines that would be considered for use in early life should be capable of inducing *in vivo* a similar pattern of T cell and DC responses in both immunologically mature and immature hosts.

Here, we assessed the T cell and DC *in vivo* activation patterns elicited by a fusion protein of two major tuberculosis antigens (Ag85B and ESAT-6) formulated in a novel adjuvant, IC31® (registered CTM and US trademark and is covered *inter alia* by international patent applications PCT/EP01/12041 and PCT/EP01/06433, both are pending or granted in several countries). The IC31® adjuvant contains a KLK peptide and a TLR-9-triggering non-CpG oligonucleotide (ODN1a) [26], [27] and confers protective efficacy in challenge models of murine tuberculosis [28], [29]. IC31®-adjuvanted Ag85B-ESAT-6 was recently shown to induce potent responses in a clinical trial [30]. We show here that this vaccine candidate elicits the exact same pattern of multifunctional CD4^+^ T cells and of focused *in vivo* DC activation in neonates as in adults.

## Results

### Induction of adult-like multifunctional neonatal CD4^+^ T cells

C57Bl/6 mice were primed at 1 week of age (i.e. at the stage of immune maturation that most closely reflect that of human neonates [16] with Ag85B-ESAT-6 (5 µg) formulated in IC31® or aluminum hydroxide (Alum, control) via the subcutaneous (s.c.) route. Mice were boosted 3 weeks later, unless indicated otherwise. Neonatal weight gain, a sensitive method of monitoring neonatal reactogenicity, was normal in each group and the incidence of local reactions (inflammatory nodules) was similar to that of adult mice (data not shown).

**Figure 1:**
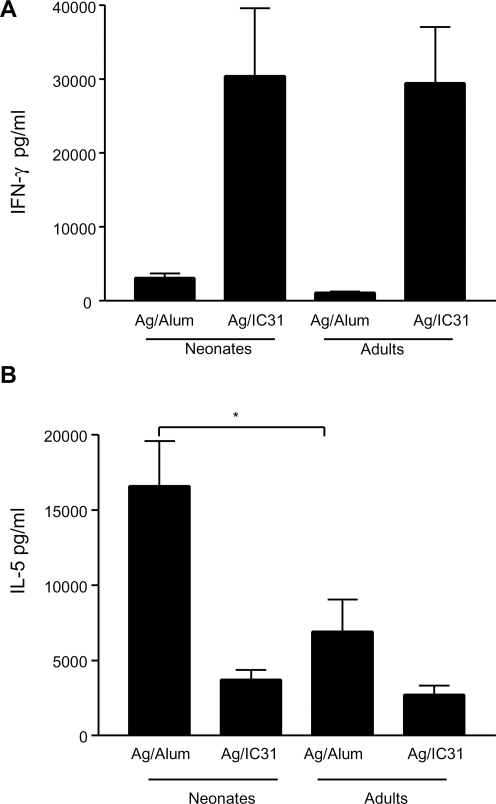
Unlike alum, the IC31® adjuvant induces adult-like Th1 cell responses in neonates. Three weeks after the second immunization, splenocytes from neonatal and adult immunized mice were restimulated with antigen for 3 days. The production of IFN-γ (A) and IL-5 (B) is represented by the mean and SD of groups of at least 6 individual mice, and is representative of 3 independent experiments. *, p<0.05 signifies differences between neonates and adult mice.

Immunization with Ag85B-ESAT-6 in Alum elicited significantly weaker IFN-γ and stronger IL-5 responses in mice primed as neonates than as adults, a Th2-preferential pattern characteristic of neonatal vaccine responses (Figure 1). In contrast, Ag85B-ESAT-6 in IC31® elicited a mirror pattern with similarly high IFN-γ responses and modest IL-5 responses in both age groups. This induction of adult-like neonatal responses was confirmed by assessing the cytokine frequency and production of IFN-γ IL-2, TNF-α and IL-17 (Table 1). Most Ag-specific CD4^+^ T cells produced TNF-α and/or IL-2 in both age groups. IFN-γ producing cells were fewer, but present at similar proportions in neonates and adults (Table 1). As previously observed in adult mice [29], only CD4^+^ T cell responses to Ag85B-ESAT-6 were elicited: Ag-specific CD8^+^ T cells were not detected by flow cytometry nor was there a decrease in IFN-γ production when CD8^+^ T cells were blocked during *in vitro* culture (data not shown). Remarkably, adult-like Th1 responses were already elicited after a single neonatal immunization in IC31® (Table 1). Adding a 3^rd^ immunization 3 weeks after the 2^nd^ dose did not further increase Ag85B-ESAT-6 specific CD4^+^ T cell responses (not shown).

**Table 1:** Ag85B-ESAT-6-specific T cell response following immunization with antigen- IC31® formulation.

	In vitro stimulation a				Ex vivo intracellular cytokine staining b (% of CD44^+^ CD4^+^ T cells)		
	Neonates	Adult		p value c	Neonates	Adult	p value
**2 doses**							
IFN-γ	292±153	213±94	ISC/10^6^ splenocytes	NS	3.8±3.2	2.9±0.3	NS
IL-2	4.9±1.3	1.2±0.5	U/ml	NS	6.8±5.6	5.1±0.6	NS
TNF-α	1.0±0.2	0.7±0.2	ng/ml	NS	7.3±5.7	5.7±0.7	NS
IL-17	7.5±2.4	9.5±5.7	ng/ml	NS	ND d	ND	
**1 dose**							
IFN-γ	17.8±1.3	6.5±0.6	ng/ml	NS	ND	ND	
IL-5	0.6±0.2	0.5±1.0	ng/ml	NS	ND	ND	

a IFN-γ secreting cells (ISC)/10^6^ splenocytes were determined after 48 hr and IL-2 (bioassay), TNF-α, IL-17, IFN-γ and IL-5 (ELISA) after 72 hr of culture.

b Following 6 hr culture with antigen and co-stimulation (CD28/CD49d), percent of cytokine-producing cells determined by flow cytometry.

c NS: p>0.05.

d ND: not done.

**Figure 2:**
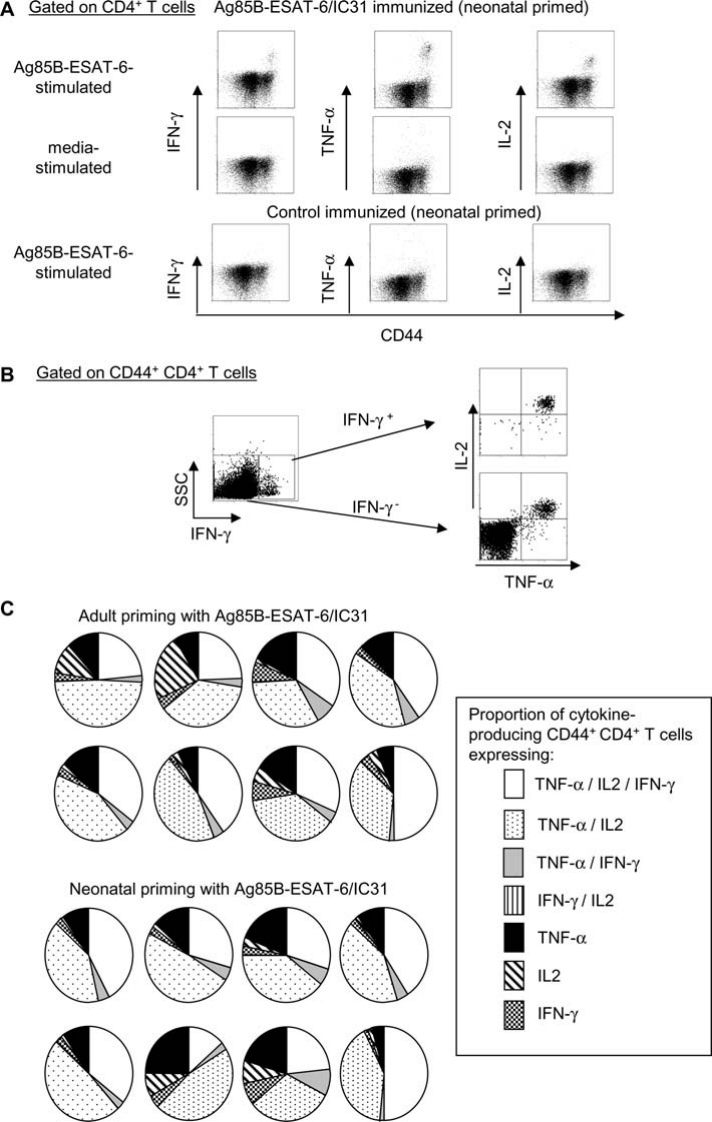
Ag85B-ESAT-6/IC31® stimulates production of multifunctional T cells in neonates and adults. The expression of IFN-γ, IL-2 and TNF-α was determined by FACS ICS on splenocytes from neonatal and adult immunized mice (A) CD4^+^ T cells, from neonates immunize with Ag/IC31® or control, were stimulated with antigen or media. Cytokine production was only detected in cells from Ag/IC31®-immunized mice stimulated with antigen. The same expression pattern was observed with splenocytes from adult immunized mice (not shown) (B) Gating combination to determine concomitant cytokine expression. Representative gating of CD4^+^ CD44^+^ T cells from Ag/IC31®-immunized mice stimulated with antigen: IFN-γ^+^ and IFN-γ^−^ populations were plotted as IL-2 versus TNF-α. (C) The concurrent expression of IFN-γ, IL-2 and TNF-α is represented as pie graphs of cytokine expression by CD4^+^ CD44^+^ T cells of individual mice, and is representative of 2 independent experiments.

To further delineate the functionality of Ag85B-ESAT-6 specific CD4^+^ T cells, the co-production of IFN-γ, TNF-α and IL-2 was assessed by FACS intracellular staining (ICS). Six weeks after boosting, IFN-γ, TNF-α and IL-2 were produced by CD44^hi^ activated/memory CD4^+^ T cells (Figure 2A). Combination gating was used to determine the cytokine production of single T cells in individual mice (Figure 2B). Although the distribution of multi-cytokine production varied as expected among individual mice, the same pattern was observed whether mice were immunized as adults or neonates (Figure 2C). Most cytokine^+^ T cells produced TNF-α/IL-2, often with IFN-γ. Remarkably, the same cytokine production pattern was observed in mice primed as neonates. Thus, neonatal immunization with IC31®-adjuvanted Ag85B-ESAT-6 was well tolerated and elicited adult-like multifunctional CD4^+^ T cell responses that markedly differ from the Th2-biased neonatal pattern induced with the Alum adjuvant.

### *In vivo* activation of neonatal and adult vaccine-targeted dendritic cells

**Figure 3:**
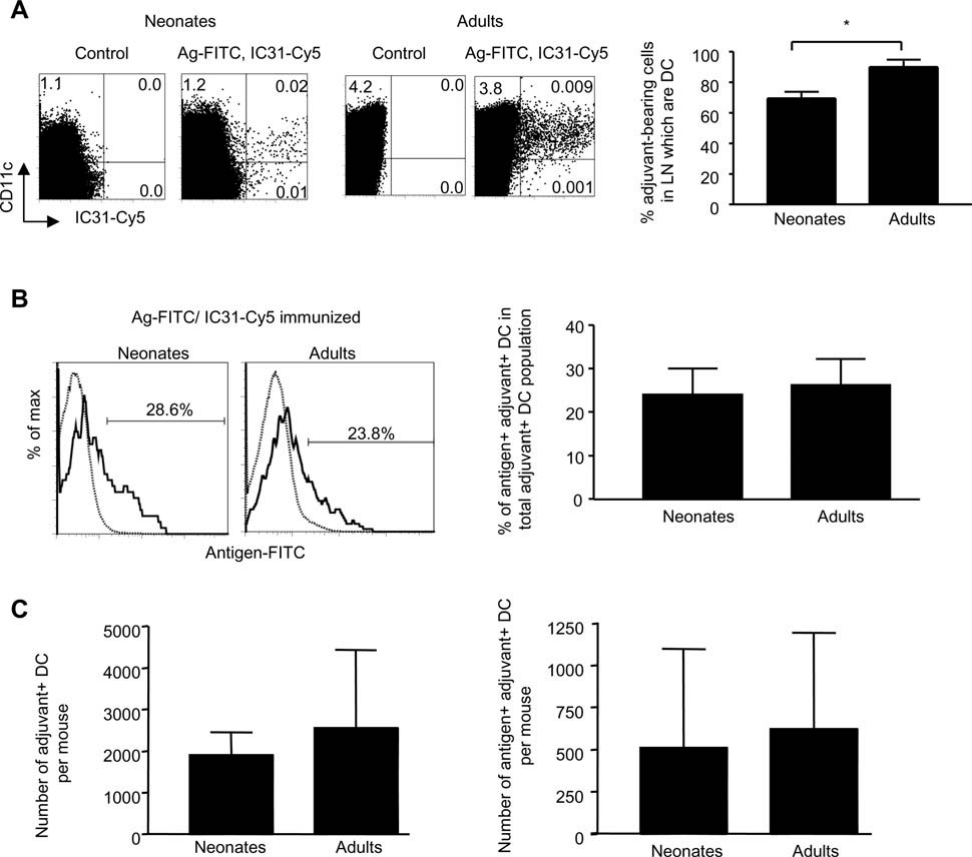
Exquisite targeting of Ag85B-ESAT-6 in neonatal and adult DC of the draining LN. Twenty-four hours after immunization, (A) the percent of IC31®-Cy5 ^+^ CD11c^+^ cells compared to all cells-bearing the adjuvant in the draining LN was determined. In a representative dot plot, the percent of cells in quadrants is shown (B) The percent of Ag85-ESAT-6^+^ IC31®^+^ DC amongst all IC31®^+^ DC was determined. In a representative histogram, presence of Ag85-ESAT-6-FITC in IC31®^+^ DC (thick line) was calculated using total DC as the control histogram (dotted line). (C) The number of IC31®^+^ and Ag85-ESAT-6^+^ IC31®^+^ DC in the draining LN was calculated. The data are expressed as mean and SD of groups of at least 5 individual mice, and is representative of 3 independent experiments. *, p<0.05 signifies differences between neonates and adult mice.

**Figure 4:**
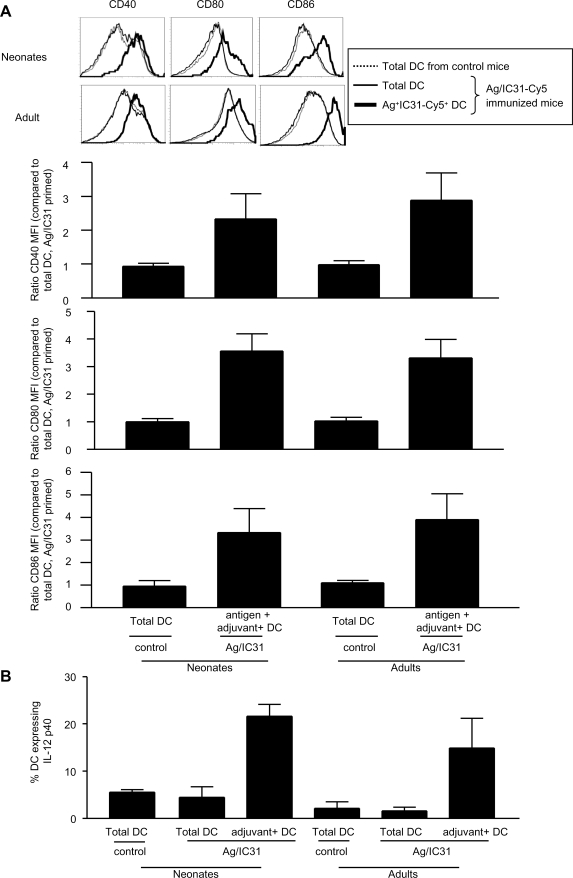
Targeted functional activation of DC in neonates and adults. Twenty-four hours after immunization, (A) the expression of CD40, CD80 and CD86 and (B) IL-12p40 by total DC and adjuvant^+^ DC was assessed. Histograms represent the expression of co-stimulation molecules by total DC in control mice (dotted line), total DC (thin lined histogram) and Ag85B-ESAT-6^+^ IC31®^+^ DC (thick lined histogram) in Ag85B-ESAT-6/IC31®-immunized mice. Data is expressed as mean and SD of groups of at least 5 individual mice, and is representative of 2–3 independent experiments.

The 2^nd^ stage of our vaccine candidate evaluation includes a detailed assessment of *in vivo* DC activation patterns. Using fluorochrome-labeled formulations to track antigen (Ag)^+^ and/or adjuvant (Adj)^+^ DC in draining lymph nodes (LN), we previously reported that IC31® induces in adult mice an exquisite DC activation that may be detected in the draining LN 24 h after priming [31]. Despite their very small size, the draining LN of 1-week-old mice were harvested 24 h after a single injection of IC31®-adjuvanted Ag85B-ESAT-6. Most Adj^+^ LN cells (∼85%) were DCs in adult LN (Figure 3A). This proportion was slightly lower in 1-week-old mice, indicating some uptake by non-DC populations. Among neonatal Adj^+^ DCs, only a minority (∼25%) were also Ag^+^ DC, as observed in adults (Figure 3B). Remarkably, the number of Adj^+^ and of Ag^+^Adj^+^ DC was similar in neonates and in adults (Figure 3C), despite the difference of LN cellularity (not shown). Assessing the surface expression of the CD40, CD80 and CD86 co-stimulation molecules indicated that IC31® only activated Adj^+^ DCs (Figure 4A) in both age groups. Remarkably, DC activation was phenotypically similar in neonates and adults. This was confirmed by the quantification of IL-12p40 expression, only visualized in Adj^+^ DC and observed at the same level in neonates as in adults (Figure 4B). Thus, a similar exquisite *in vivo* DC activation pattern was elicited by IC31®-adjuvanted Ag85B-ESAT-6 both in adults and neonates.

**Figure 5:**
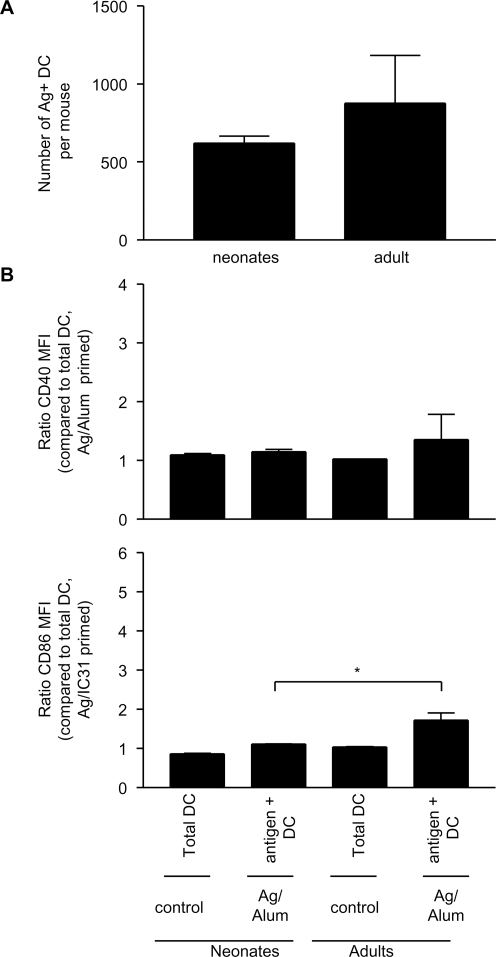
Alum induces similar uptake of antigen, but a slight activation of antigen^+^ DC in adult compared to neonates. Twenty-four hours after immunization, (A) the number of antigen ^+^ DC and (B) the expression of CD40 and CD86 by total DC and antigen^+^ DC were assessed. The data are expressed as mean and SD of groups of at least 5 individual mice, and is representative of 2 independent experiments. *, p<0.05 signifies differences between neonates and adult mice.

As Ag85B-ESAT-6 in Alum did not elicit the same pattern of T cell responses in neonates and in adults (Figure 1), potential differences in the pattern of *in vivo* DC activation elicited by immunization in Alum were investigated. As aluminium salts could not be fluorescently labeled, LN cells harvested 24 h after immunization were gated on Ag^+^ DC. Remarkably, the number of Ag^+^ DC was similar in adults and neonates (Figure 5A), and similar (∼500 cells) to that elicited by IC31® (Figure 3). In adults, a slightly higher expression of CD86, but not of CD40, was observed on Ag^+^ DC (Figure 5B). This partial activation of adult Ag+ DCs did not translate into any increase of IL-12 p40 expression and was not observed in neonates (Figure 5B and data not shown) despite the efficient targeting of neonatal DC.

### Protective efficacy of novel vaccines against mycobacterial infection

**Figure 6:**
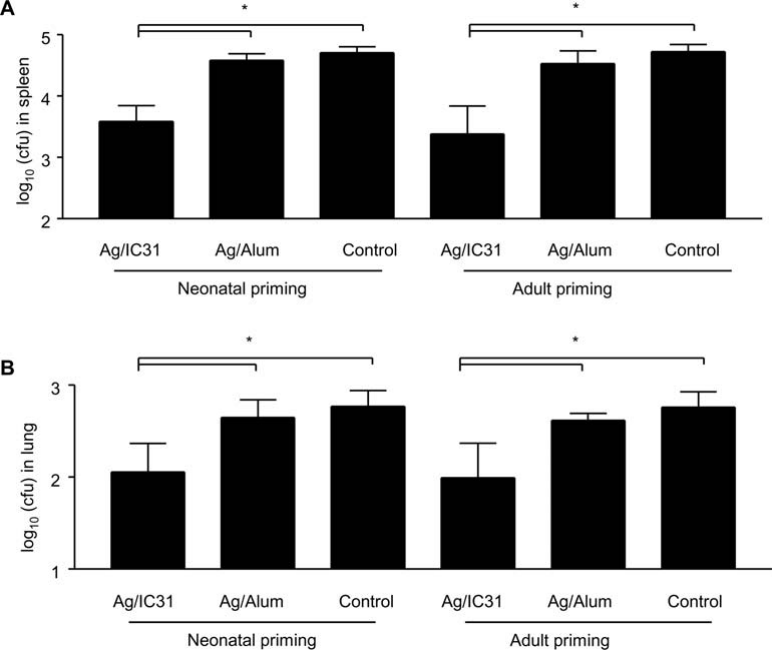
Ag85B-ESAT-6/IC31® protects neonates and adults from mycobacterial infection. Immunized neonatal and adult mice were challenged with *Mycobacterium bovis* BCG i.v. The CFU in spleen (A) and lung (B) (mean±SD, n = 6–8/group) were determined four weeks later, and is representative of 2 independent experiments. *, p<0.05, differences between IC31® and Alum or control were significant.

The 3^rd^ stage of our TB vaccine evaluation includes a mycobacterial challenge model. Six weeks after the second immunization with IC31®-adjuvanted Ag85B-ESAT-6, mice were challenged i.v. with *Mycobacterium bovis* BCG. In accordance with CD4^+^ T cell responses, Ag85B-ESAT-6 did not confer any protective efficacy when formulated in Alum, as the number of CFU in spleen and lungs was as high as in control mice regardless of age at immunization (Figure 6A,B). In contrast, significantly lower bacterial counts were recovered from mice immunized with IC31®-adjuvanted Ag85B-ESAT-6 either as adults or as neonates (Figure 6A, B).

## Discussion

Conventional vaccine formulations elicit Th2-biased CD4^+^ T cell responses when used in early life and specific requirements have to be met for vaccine formulations and/or adjuvants to successfully elicit adult-like Th1 neonatal responses [32], [33]. We show here that a novel subunit vaccine currently in clinical development against TB meets all the predefined preclinical criteria predicting adult patterns of human immune responses after administration in early life.

When mice were immunized with Ag85B-ESAT-6/IC31®, an extensive evaluation of CD4^+^ T cell responses failed to identify any difference between antigen-specific responses elicited in neonates and adults. This was demonstrated for a large panel of Th1 cytokines including TNF-α, IFN-γ, IL-2 and IL-17, which were assessed in the supernatant of *in vitro* restimulated T cells and/or through the number of cytokine-producing cells. An adult-like pattern of responses was also observed following the quantification of multifunctional CD4^+^ T cells secreting 2–3 distinct Th1 cytokines. Whether multifunctional T cells do play a direct role in the control of Mtb is yet unknown. However, that such complex patterns are conserved even when immunization takes place in immature neonatal hosts strongly suggests that Ag85B-ESAT-6/IC31® mediated signals fully overcome the limitations of innate and adaptive neonatal immunity. This achievement may not be accounted for by ongoing immune maturation, as an adult pattern of CD4^+^ T cell responses was already observed after a single immunization of 1-week-old mice. It may also not be ascribed to immunogenic properties of Ag85B-ESAT-6, as its use with Alum generated markedly biased Th2 neonatal responses. Thus, the induction of multifunctional CD4^+^ Th1 cells both in immature and mature hosts reflects the potent Th1-driving capacity of IC31®. Yet, this was not associated with excess inflammatory reactions – meeting another important criterion for considering the use of vaccine candidates in neonates. In contrast to the neonatal use of a number of other immunomodulators or novel adjuvants [18], [21], [32], [34] (and not shown), we observed neither systemic nor local reactogenicity following s.c. injection of Ag85B-ESAT-6/IC31® in neonates.

Immune responses elicited by Ag85B-ESAT-6/IC31® conferred significant protection against a non-lethal mycobacterial challenge: following BCG infection, mycobacterial counts were significantly reduced in the lungs and spleens of mice immunized with Ag85B-ESAT-6/IC31®, as compared to the use of Alum. This reduction was similar regardless of age at immunization, indicating again no influence of the stage of immune maturation on vaccine-induced protection. Acknowledging the fact that a non-lethal BCG challenge is only a proxy of Mtb infection, neonates were primed with Ag85B-ESAT-6/IC31®, boosted and submitted to an aerosol Mtb challenge as described [29]. Logistic limitations only allowed us to run a single experiment, which concluded to a significant reduction of bacterial counts in immunized compared to naïve mice (not shown). Although this preliminary observation will needs to be confirmed in the future, it adds further evidence to our conclusion that the use of IC31® as an adjuvant in early life vaccines does overcome neonatal immune limitations.

The main stage of our preclinical neonatal vaccine evaluation platform relies on the assessment of *in vivo* DC activation. Indeed, the unavailability of validated surrogates of protective responses frequently limits the predictive capacity of preclinical models: immunological studies would not include yet unidentified cytokines/patterns of responses, whereas protective mechanisms may differ across species. Being at the intersection of innate and adaptive immunity, DC targeting and activation is a most sensitive marker of differences in responses to pathogen-associated molecular patterns (PAMPS)-mediated signaling [35], [36]. In this report, Ag85B-ESAT-6 was used with Alum and IC31®, two adjuvants whose biochemical/signaling properties markedly differ. Remarkably, only a small but similar number of Ag^+^ LN DC were identified in the draining LN 24 h after the injection of IC31®- or Alum-adjuvanted Ag85B-ESAT-6. Factors that control DC antigen uptake/migration were thus not modulated by the use of two adjuvants markedly differing in their Th1 (IC31®) or Th2 (Alum) properties.

Both innate and adaptive immunity markedly differ in early life [18], [33]. Consequently, both human and murine neonatal DC differ from their adult counterparts in their response capacity to different PAMPS [20], [37]–[39]. The direct assessment of vaccine-targeted neonatal DC in the minute draining LN of 8-day-old mice was technically challenging. However, this was required to define the influence of immune maturation on the type of vaccine-targeted cells, their numbers and their state of activation following immunization. The observation of a similar number of Ag^+^ LN DC in neonates and in adults indicates that DC antigen uptake/migration capacities are already fully functional at 7 days of age, even when adjuvants as weak as Alum are used. These similarities are quite remarkable given the differences in the numbers of DC present in neonatal and adult mice [37], [38]. They do not result from a more mature LN microenvironment [18] as a similar distribution of vaccine-targeted cells was observed in the spleen following i.p. immunization (not shown). Thus, neither the excess Th2 responses of neonates injected with Alum or the strong Th1 responses following the injection of IC31® result from differences in the numbers of Ag^+^ DC.

The neonatal use of IC31® elicited not only an adult-like number of Ag^+^ cells but also their adult-like activation pattern. This activation was restricted to Adj^+^ cells, whether DC were also Ag^+^ or not, as previously observed in adults [31]. Both phenotypic maturation and IL-12p40 expression were adult-like, indicating that neonatal DCs responded to IC31®-generated signals within 24 h of immunization. As IC31® is known to signal through TLR-9, this observation is in accordance with reports by us [40], [41] and others [37], [39], [42], [43] that TLR-9 signals are readily recognized by neonatal murine and human DC.

In conclusion, this is the first report that a novel subunit vaccine against TB may elicit adult-like multifunctional and protective CD4^+^ T cell responses through the induction of an adult pattern of *in vivo* DC activation in neonates. We cannot ensure that this will be sufficient to confer adult-like protective capacity to this subunit vaccine in human neonates. But this preclinical evaluation fully supports including this novel vaccine among the most promising novel infant TB vaccines.

## Materials and Methods

### Mice

Specific pathogen-free C57BL/6 mice (7 days of age (1-week-old, neonates) or 8–12 weeks old (adult), Charles River) were bred and kept under specific pathogen-free conditions in the university zootechnology unit. Mice were immunized at the base of the neck using the subcutaneous (s.c.) route. Mice received two (three weeks apart) or one dose of the formulations for analysis of T cell function and challenge studies. For analyses of *in vivo* DC activation, the axial, brachial and auricular lymph nodes were harvested 24 hours following a single immunization. Manipulations were conducted according to Swiss and European guidelines and experiments approved by the Geneva Veterinary Office.

### Antigen, Adjuvants, TLR ligands and immunization

Recombinant Ag85B-ESAT-6 was prepared as described [28] and coupled with fluorescein (Sigma, St. Louis, Mo.) or AlexaFluor 647 (Invitrogen Basel, Switzerland). IC31®, consisting of KLK (NH_2_-KLKL_5_KLK-COOH) and ODN1a (phosphodiester backbone ODN, 5′- ICI CIC ICI CIC ICI CIC ICI CIC IC-3′), including TAMRA-coupled KLK and Cy5-coupled ODN1a, was produced as described [26]. Vaccines were formulated by absorbing antigens (5 or 15 µg) with IC31® (100 nmol KLK/4 nmol ODN1a) in 10 mM Tris–HCl/270 mM sorbitol buffer (pH 7.5–8) or Al(OH)_3_ (Alum, gift of Chiron Vaccines). The adult dose of Al(OH)_3_ (1 mg) was weight-adjusted to 0.25 mg for immunization of 1-wk-old mice respectively, as previously experimentally defined [22]. The buffer was used for control immunization.

### Determination of T cell responses

Three weeks after immunization (either second of two doses or a single dose), splenocytes were cultured with Ag85B-ESAT-6 (5 µg/ml) or medium alone. For determination of cytokine by ELISA or bioassay, supernatants collected after 72 h for quantification by ELISA of IFN-γ and IL-5 [17], TNF-α (BD Biosciences, San Diego, CA), IL-17 (R&D Systems, Abingdon, UK) and by bioassay IL-2 [44]. Under these conditions, blocking studies with antibodies against CD4 (clone GK1.5) and CD8 (clone H35-17.2) were undertaken. The antigen-specific IFN-γ-secreting T cells were quantified by ELISPOT, using Ag85B-ESAT-6 (2 µg/ml) or media alone for 48 h [45]. For determination of cytokine expression and multi-functional T cells by intracellular staining (ICS), splenocytes were cultured with Ag85B-ESAT-6 (5 µg/ml) or medium alone as well as anti-CD28 and anti-CD49d antibodies (BD Pharmingen) for 1 hour, before the addition of Brefeldin A and monensin (Sigma). Following an additional 6 hour incubation, cells were stained with conjugated antibodies against CD4 (clone GK1.5), CD8α (clone 53-6.7) and CD44 (clone IM7) and then fixed and permeabilized with BD Cytofix/cytoperm solution. Cells were stained with conjugated antibodies against IFN-γ (clone XMG1.2), TNF-α (clone MP6-XT22), IL-2 (clone JES6-5H4) (BD Pharmingen) and IL-17 (clone eBio17B7)(eBioscience, San Diego, CA). Each sample was acquired on the FACSAria cytometer (BD Biosciences) and data were analyzed using the FlowJo Software (Tree Star, Ashland, OR) and gating combination shown in Figure 2.

### Dendritic cell preparation, cell staining and flow cytometry

CD11c^+^ DC from draining LN, 24 hours after immunization, were prepared by magnetic selection (Miltenyi Biotec, Bergisch-Gladbach, Germany) as described [31]. Cells were pre-incubated with rat anti-CD16/32 mAb (2.4G2 clone), then stained with conjugated antibodies against CD11c (HL3 clone), CD80 (16-10A1 clone), CD11b (M1/70 clone), CD8 (53-6.7 clone), isotype controls (BD Pharmingen), CD86 (GL1 clone) (Biosource International, Camarillo CA), CD11c (N418 clone), CD40 (FGK45 clone) (produced in house). Cells were further stained with streptavidin-PE or streptavidin-PECy7 (BD Pharmingen). Each sample was acquired on the FACSCalibur or FACSAria cytometers and data were analyzed using the CellQuest Software (BD Biosciences) or the FlowJo Software. Results were expressed as the ratio of Mean Fluorescence intensity (MFI) compared to controls in order to allow objective comparisons between experiments. IL-12p40 was detected by ICS as described [31].

### Mycobacterial challenge

Six weeks after the second immunization, mice were infected i.v with 10^7^ CFU of *Mycobacterium bovis* BCG Danish 1331. Four weeks p.i., mice were sacrificed and spleens and lungs homogenized for bacterial enumeration. Individual organs were plated in serial dilutions onto Middlebrook 7H11 agar and incubated for 3 weeks at 37°C prior to counting the number of CFU.

### Statistical analysis

Statistical analyses of the results obtained in various experimental groups were performed with the Mann-Whitney U test (two groups) or ANOVA with Tukey test (more than two groups). Differences with probability values of >0.05 were considered insignificant.
